# Supplementary material for: A Proteomic Analysis of Detergent-Resistant Membranes in HIV Virological Synapse: The Involvement of Vimentin in CD4 Polarization
Source: Viruses. 2023 May 28;15(6):1266. doi: 10.3390/v15061266 (PMC10304898; doi:10.3390/v15061266)
Supplement: Supplementary file 1 [file viruses-15-01266-s001.zip › viruses-2369148-supplementary.pdf]

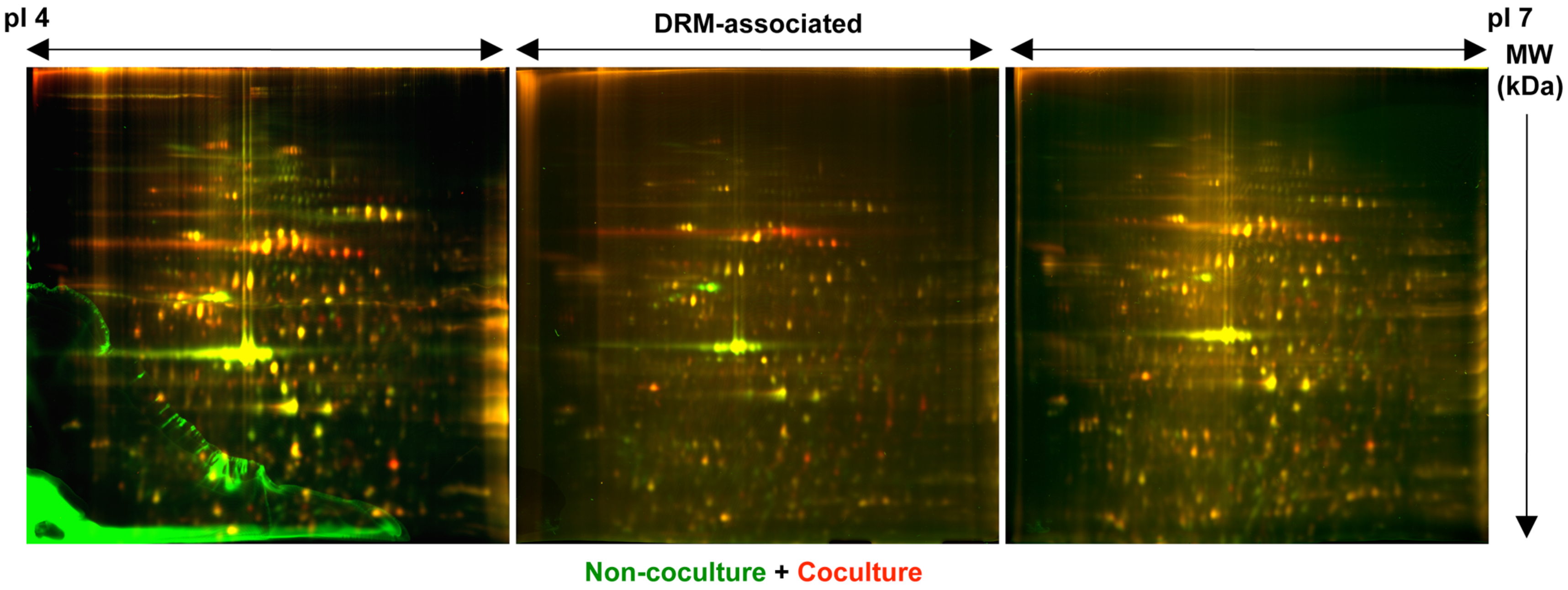

Supplementary Figure S1. 2-D DIGE analyses of the DRM proteins isolated from coculture and non-coculture samples of HIV-1-infected and uninfected Jurkat cells. The methods for 2-D DIGE analysis were described in the legend for Figure 2. Additional three gel images (in 6 independent experiments) are shown.

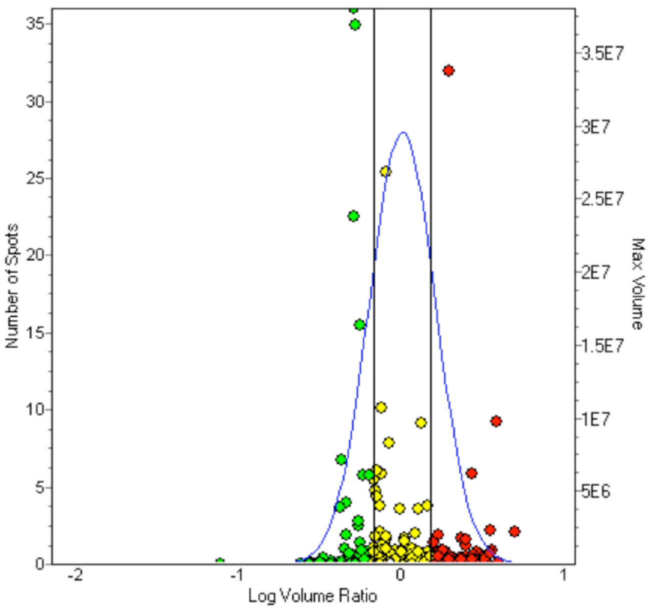

Supplementary Figure S2. Distribution of the spot volume ratios of 2-D DIGE. The frequency distribution of volume ratios for spots detected in the 2-D DIGE images shown in Figure 2C is illustrated by the normalized model, depicted as a blue curve. The volume of each protein spot, represented as a single data point, is plotted on the right axis. Red spots represent proteins with a greater-than-1.5-fold increase in the cocultured samples, while green spots represent proteins with a greater-than-1.5-fold decrease.

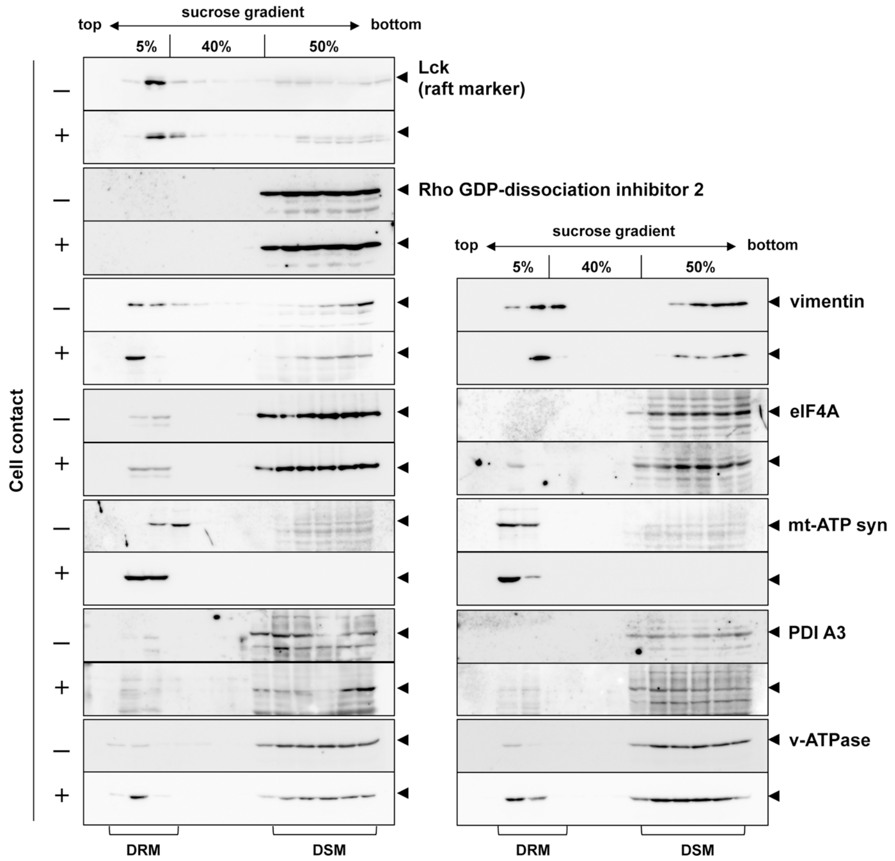

Supplementary Figure S3. Membrane flotation of the identified molecules in this study. Jurkat cells were infected with HIV-1 and incubated with uninfected Jurkat cells, and the cell coculture was lysed with Triton X-100 on ice. Following membrane flotation centrifugation, the gradient fractions were analyzed by western blotting with anti-Lck, anti-Ly-GDP-dissociation inhibitor, anti-vimentin, anti-eIF4A, anti-mt-ATP synthase, anti-ERp57/PDI A3, and anti-v-ATPase antibodies. Additional blot images were shown.
